# Assessment of Cannabinoids Agonist and Antagonist in Invasion Potential of K562 Cancer Cells

**DOI:** 10.29252/.23.2.153

**Published:** 2019-03

**Authors:** Fatemeh Gholizadeh, Mohammad Hossein Ghahremani, Shima Aliebrahimi, Amir Shadboorestan, Seyed Nasser Ostad

**Affiliations:** 1 Department of Toxicology and Pharmacology, Faculty of Pharmacy and Poisoning Research Center, Tehran University of Medical Sciences, Tehran, Iran;; 2 Department of Cellular and Molecular Biology, School of Biology, College of Science, University of Tehran, Tehran, Iran

**Keywords:** AM251, Cannabinoid receptor, Leukemia, Matrix metalloproteinases, WIN 55212-2

## Abstract

**Background:**

The prominent hallmark of malignancies is the metastatic spread of cancer cells. Recent studies have reported that the nature of invasive cells could be changed after this phenomenon, causing chemotherapy resistance. It has been demonstrated that the up-regulated expression of matrix metalloproteinase (MMP) 2/MMP-9, as a metastasis biomarker, can fortify the metastatic potential of leukemia. Furthermore, investigations have confirmed the inhibitory effect of cannabinoid and endocannabinoid on the proliferation of cancer cells *in vitro* and *in vivo*.

**Methods:**

In the present study, the inhibitory effect of WIN 55212-2 (a CB1/CB2 receptor agonist) and AM251 (a selective CB1 receptor antagonist) on K562 cells, as a chronic myelogenous leukemia (CML) model, was evaluated using MTT and invasion assay. Expressions of MMP-2 and MMP-9 were then assessed by Western blot analysis.

**Results:**

The data obtained from MTT assay showed that WIN 55212-2 could attenuate cell proliferation; however, AM251 was less effective in this regard. Our results showed that WIN 55212-2 considerably reduced cancer cell invasiveness, while AM251 exhibited a converse effect. Moreover, CB1 activation resulted in decreased expression of MMP-2 and MMP-9.

**Conclusion:**

Our findings clarifies that CB1 receptors are responsible for anti-invasive effects in the K562 cell line.

## INTRODUCTION

Chronic myelogenous leukemia (CML) is an abnormal proliferation of myeloid series accounting for 15-20% of all leukemia with a global incidence of approximately 1-2 in 100,000 adults per year. Despite the occurrence of this disease at any age, its incidence has a correlation with age. Given the high frequency of CML in men, the disease is commonly diagnosed in the sixth decade of life^[^^1^^,^^2^^]^. Studies have corroborated that in 95% of CML patients, reciprocal translocation t(9;22)(q34;q11) leads to the generation of the *bcr-abl* oncogene on truncated chromosome 22, known as Philadelphia chromosome. Moreover, it produces a constitutively active tyrosine kinase that in turn promotes the development of CML^[^^2^^,^^3^^]^. 

Metastasis of invasive tumor cell is a prominent feature of cancer in most types of solid tumors. The procedure of metastasis is the dissemination of tumor cells from the primary tumor mass to other tissues. In summary, this process involves sequential processes including invasion that enables tumor cells to departure from the primary tumor, intravasation of metastatic cells into the blood and lymphatic circulation system, extravasation, and finally tumor cell colonization and angiogenesis to form the metastatic injury^[^^4^^-^^6^^]^. 

For intravasation and extravasation processes, degradation of the collagen-rich extracellular matrix (ECM) and basement membrane is necessary^[^^7^^]^. In this regard, matrix metalloproteinases (MMPs) have been considered as primary proteases responsible for ECM disintegration during tumor metastasis^[^^8^^]^. The MMP family of zinc endopeptidases consists of at least 26 proteases and is subdivided into four classes: collagenases, gelatinases, stromelysins, and matrillysins. Different cells such as endothelial cells, leukocytes, macrophages, fibroblasts, and tumor cells can produce MMPs. Accumulated evidence implies that MMPs, the Mr 72,000 type IV collagenase (MMP-2) and the Mr 92,000 type IV collagenase (MMP-9), play an important role in a variety of pathologies such as tumor angiogenesis and metastasis^[^^9^^-^^13^^]^. 

During the past decades, the 21-carbon terpenophenolic compounds, cannabinoids, extracted from *Cannabis sativa* (marijuana, hemp plant) have been identified with a wide spectrum of pharmacological effect. They mimic the effects of endogenous cannabinoids named as endo-cannabinoids^[^^14^^]^. The CB1 and CB2 are two main cannabinoid-specific receptors with different tissue expression patterns. Studies have indicated that CB1 is predominantly expressed in central nervous system and peripheral tissues; instead, the CB2 receptor is exclusively expressed in the immune system. These G protein-coupled receptors have several physiological functions in the processes of pain and anxiety. Desensitization process usually occurs due to the prolonged exposure of agonist with the receptor. This event is modulated by receptor degradation or down-regulation. Recently, particular attention has been focused on anticancer properties of cannabinoids. To date, several mechanisms have been proposed, of which the inhibition of invasion and metastasis is attributed to their anti-neoplastic activity^[^^15^^-^^17^^]^.

 Here, we aimed to investigate the role of the CB1 cannabinoid receptor in the proliferation and invasion potential of K562 cell line. These cells have been characterized by CB1 expression (www.proteinatlas. org), while CB2 expression has not been detected^[^^18^^]^. 

## MATERIALS AND METHODS

**Chemical reagents and antibodies**

WIN 55212-2 (a potent agonist of CB1) was purchased from Cayman Chemical Company (Ann Arbor, MI, USA), and AM251 (a selective antagonist of CB1) was obtained from Sigma-Aldrich (St. Louis, MO, USA). For Western blotting, antibodies MMP-2 and MMP-9 were procured from Abcam (Cambridge, UK), β-actin from Santa Cruz Biotechnology (Santa Cruz, CA, USA), and goat anti-mouse/anti-rabbit IgG secondary antibody from Bio-Rad (Hercules, CA). 3-(4,5-dimethylthiazol-2-yl)-2,5-diphenyltetrazolium bromide (MTT) and dimethyl sulfoxide (DMSO) were provided by Sigma-Aldrich. PVDF membrane and ECL kit were purchased from Roche (Germany).

**Cell culture**

The human K562 (CML) cells purchased from Pasture Institute of Iran (Tehran) were maintained in RPMI-1640 medium (Biosera, East Sussex, UK) supplemented with 10% heat-inactivated fetal bovine serum (FBS; Gibco, USA), 1% penicillin-streptomycin (Biosera) in a 5% CO_2_ incubator at 37 °C.

**MTT assay**

K562 cells were seeded at the density of 5 × 10^4^ cells/well in a 24-well plate. After 12 h, the cells were treated with various concentrations of WIN 55212-2 (0.1-6.4 μM) and AM251 (0.1-0.5 µM) for 48 h. Afterward, 200 μL MTT (5 mg/mL in PBS) was added to each well and incubated at 37 °C for an additional 4 h. Following centrifugation, the supernatants were aspirated, and 600 μL DMSO was added as a solvent to reduce the MTT dye. Finally, the absorbance was measured using an ELISA microplate reader (Anthos, UK) at 570 nm with a reference wavelength of 690 nm. Cell viability was obtained by the following equation:

%Cell viability = (OD_test_/OD_control_) ×100

**Invasion assay**

For evaluation of invasion capacity of K562 cells, Matrigel invasion assay was performed according to the modified Boyden chamber method. The assay was done using transwell inserts (Corning, USA; diameter, 6.5 mm; pore size, 8 µm) preloaded with 32 µL of diluted (1:4) matrigel in serum-free medium and incubated at 37 °C for 6 h to form a semisolid matrix. K562 cells were washed three times with a culture medium containing 1% FBS. Then the cells were treated with indicated concentration of WIN 55212-2 (3 μM) and AM251 (0.2 µM) and seeded on the upper chamber (1 × 10^5^ cells/well in serum-free medium). Medium supplemented with 20% FBS was placed in the lower chamber to serve as a chemo-attractant in 24-well plates. After incubation for 48 h, non-migrated cells on the top surface were gently wiped with a cotton swab. Subsequently, the invaded cells on the lower surface of the insert were fixed with cold methanol for 10 min, stained with H&E and counted in ten randomly selected fields per each membrane by a light microscope. A non-treated group was applied as a control. 

**Western blotting**

For Western blot analysis, 5 × 10^5^ cells/well were seeded into a 6-well plate, followed by incubation at 37 °C overnight. The cells were then treated with WIN 55212-2 (3 μM) and AM251 (0.2 µM) for 48 h. Thereafter, the cells were washed with PBS and lysed at 4 °C in a lysis buffer (62.5 mM Tris, pH 6.8, 2% sodium dodecyl sulphate [SDS], 10% glycerol, 50 mM dithiothreitol, and 0.25% [w/v] bromophenol blue). Protein samples (30 µg) quantified by Lowry protein assay were subjected to 8% SDS-PAGE and transferred onto PVDF membrane. Membranes were then blocked with 5% bovine serum albumin (BSA) in TBST (Tris-buffered saline, 0.1% Tween 20) buffer and incubated with the following primary antibodies: MMP-2 (1:500 dilution), MMP-9 (1:1000 dilution), and β-actin (1:5000 dilution) with agitation at 4 °C overnight. After being washed with TBST buffer three times for 30 minutes at room temperature with agitation, horseradish peroxidase-conjugated goat anti-mouse IgG or goat anti-rabbit IgG (1:5000 dilution) was used. Finally, ECL system was used for visualization of membranes. β-actin served as an internal control.

**Statistical analysis**

Results were presented as mean ± SD. One-way analysis of variance (ANOVA) followed by Tukey's post hoc test was used to compare differences between various treatment groups using GraphPad Prism 5.01 (San Diego, CA). Statistical probability of <0.05 was considered significant. 

## RESULTS

**Cytotoxic effect of** **WIN 55212-2 and AM251** **on K562 cells**

**Fig. 1:**
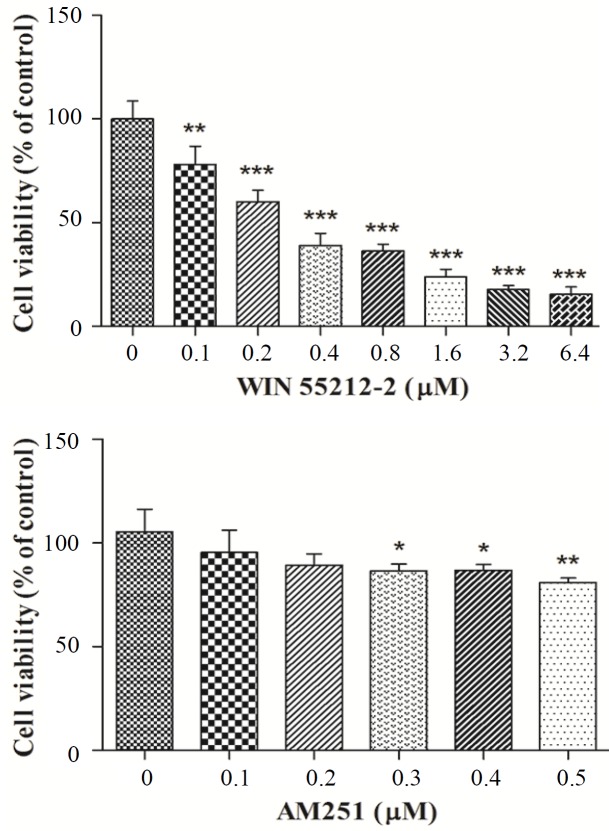
The cytotoxic effect of WIN 55212-2 and AM251 on K562 cell viability. Cells were treated with various concentrations of WIN 55212-2 and AM251 for 48 h. ^*^p < 0.05, ^**^p < 0.01, and ^***^p < 0.001 as compared with the controls

The cytotoxic activity of various concentrations of WIN 55212-2 and AM251 on K562 cells was evaluated using MTT assay. Our results indicated that treatments with WIN 55212-2 abolished cell growth in a dose-dependent manner with an IC_50_ value of 2.7 ± 0.47 µM. However, AM251 at micromolar concentrations did not mediate a remarkable inhibition of K562 cell proliferation, especially at the treated dose used in invasion assay (Fig. 1).

**Invasion analysis of** **K562 cells** **after exposure to** **WIN 55212-2 and AM251**

**Fig. 2:**
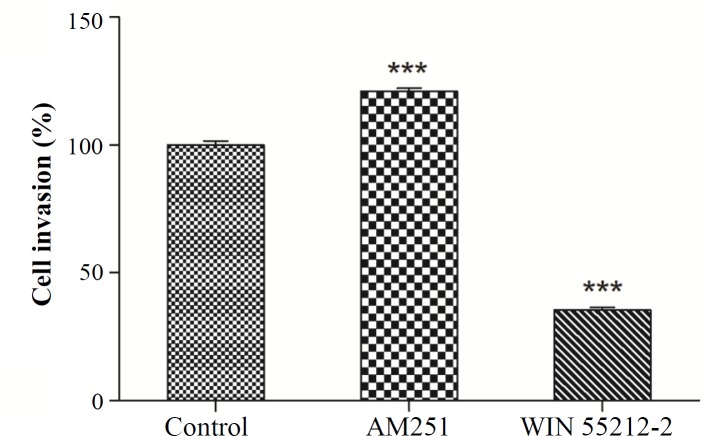
K562 cells were treated with 3 μM WIN 55212-2 and 0.2 µM AM251 for 48 h. Invasion assay was performed by using the Boyden chamber method. ^***^p < 0.001 as compared with the controls

We analyzed the inhibitory effect of WIN 55212-2 and AM251 on invasion potency in K562 cells. The cells were treated with either WIN 55212-2 (3 μM) or AM251 (0.2 µM) for 48 h. As shown in Figure 2, treatment of K562 cells with AM251 could significantly increase invasion, whereas WIN 55212-2 suppressed invasiveness compared with the untreated cells (*p *< 0.001).

**Western blotting assay for MMP-2 and MMP-9 expressions**

**Fig. 3:**
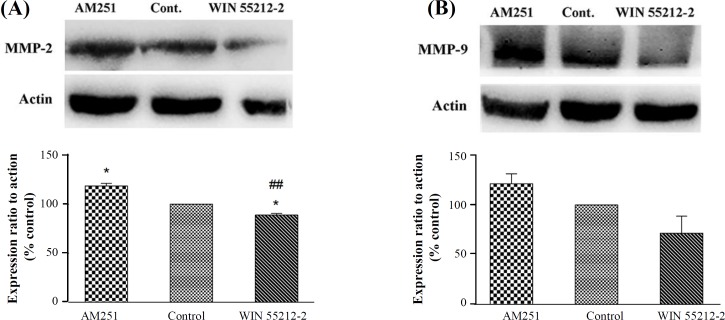
Expression of (A) MMP-2 and (B) MMP-9 after treatment with 3 μM WIN 55212-2 and 0.2 µM AM251 by Western blot analysis. Data were expressed as mean ± SEM. ^*^p < 0.05 as compared with the control group. ^##^*p *< 0.05 as compared with the AM251 group

Western blot analysis was used to evaluate whether exposure to CB1 receptor agonist and antagonist changes the expression of MMP-2 and MMP-9 involving in K562 invasiveness. Thus, K562 cells were treated with 3 µM WIN 55212-2 and 0.2 µM AM251 for 48 h. Figure 3 indicates that in cells treated with AM251, the expression of MMP-2 and MMP-9 increased, whereas WIN 55212-2 in comparison to the control group decreased the expression of MMP-2 and MMP-9, though no significant difference was observed in the case of MMP-9 expression for tested compounds (*p* > 0.05).

## DISCUSSION

Cannabis is an active ingredient of a one-year-old plant called *C. sativa*. Marijuana is a hallucinogenic substance, which is produced from the extract of dried leaves and flowers of this plant. More than 60 aromatic hydrocarbons, known as cannabinoids, are extracted from *C. sativa*, which act mainly via two receptors (CB1 and CB2). For sometime now, many investigations have been conducted on the anticancer effects of cannabinoids. Their results indicated that these compounds decrease proliferation, invasion, and angiogenesis in various cancers^[^^19^^,^^20^^]^. Additionally, several studies have shown that cannabinoid functions are dedicated in tumor cells compared to non-tumor cells, made them suitable for cancer treatment^[^^21^^-^^23^^]^. It has been reported that not only cannabinoids could reduce cancer cell growth in glioma but also could protect normal glial cells, astroglia, and oligodendroglial junction against CB1-induced apoptosis^[22]^. In the present study, our MTT assay showed that CB1 agonist WIN 55212-2 exhibited anti-proliferative activity against K562 cells, which is in line with the result obtained by Sarfaraz *et al.*^[^^24^^]^. They observed that the treatment of LNCap cells (prostate cancer) with WIN 55212-2 reduced tumor cell proliferation in a dose- and time-dependent manner without cytotoxicity against PrEC cells (normal prostate epithelial cells)^[^^24^^]^. With regard to AM251, a selective CB1 antagonist, it was less effective in terms of cytotoxic activity at concentrations of 0.1-0.5 µM. It has been shown that AM251 at concentrations 0.1-10 µM did not significantly affect cell viability in MIA PaCa-2 cells^[^^25^^]^. However, inhibition of CB1 by AM251 led to a decrease in the viability of A375 cells at 0.1-50 µM concentrations^[^^26^^]^.

 Cancer cell invasion belongs to crucial events in tumor growth and metastasis. The CB1 has been known as a suppressor of cell invasion and migration in many types of cancer, including breast, cervical, lung, prostate, and colon cancers^[^^27^^,^^28^^]^. A previous study has demonstrated that cannabinoid treatment result in the inhibition of gastric cancer cell invasion as well as the down-regulation of VEGF-A and MMP-2 expression mediated through the cannabinoid receptors CB1 and CB2^[^^29^^]^. 

 According to our results, WIN 55212-2 blocked cell invasion through the CB1 receptor. Conversely, we showed that AM251 increased the invasion of K562 leukemia cells, confirming a pivotal role of the CB1cannabinoid receptor in K562 cell invasion. It has also been demonstrated that the administration of AM251 increased the invasion potential of breast cancer^[^^30^^]^. Consistent with this finding, Nithipatikom and colleagues^[^^31^^]^ found that WIN 55212-2 in a concentration-dependent manner was able to inhibit DU-145 and PC-3 cell invasion by modulating the CB1 receptor pathway.

 As mentioned previously, MMP enzymes are the main mediator of cancer cell invasiveness. Meanwhile, various studies have evaluated the cannabinoid effects on the expression of MMPs, implying their direct effect on invasion. In the present study, the decreased expressions of MMP-2 and MMP-9 were observed following WIN 55212-2 treatment; however, AM251 increased the expression of MMP-2 and MMP-9. This finding is concurrent with our invasion assay. It has been revealed that exposure to WIN 55212-2 decreased markedly MMP-9 activity and expression both in hepatocellular carcinoma and non-small cell lung cancer cells^[^^32^^,^^33^^]^. Similarly, WIN 55212-2 treatment caused the down-regulation of MMP-2 in the cervical cancer cell in a dose-dependent manner^[^^34^^]^. 

 Several studies on a variety of cancer cell lines have confirmed that the expression pattern of cannabinoid receptors are involved in their anticancer effects^[^^35^^]^. Although treatment with cannabinoid agonists augment the expression of the CB1 receptor in various types of cancer cell lines, they lead to a reduction of receptor expression in normal tissue^[^^36^^]^. This difference in expression may be due to the existence of CB1a and CB1b (splice variants of CB1) in malignant and normal cells^[^^37^^]^. Furthermore, it should be mentioned that the aberrant expression of CB1 receptor is associated with lower survival in prostate and pancreatic cancer^[^^38^^]^. 

 In summary, the findings of this study indicated that treatment of K562 cells with WIN 55212-2 decreased cell viability in a dose-dependent manner. Nevertheless, AM251 at micromolar concentrations did not have remarkable cytotoxicity activity. Meanwhile, in cells treated with AM251, the invasion potential significantly increased, whereas WIN 55212-2 abolished invasiveness via the CB1 receptor. Moreover, CB1 activation resulted in a decreased expression of MMP-2 and MMP-9. The results disclosed here demonstrate the potential of WIN 55212-2 to attenuate the invasion of leukemic cells.

## References

[1] Siegel RL, Miller KD, Jemal A (2015). Cancer statistics, 2015. CA: a cancer journal for clinicians.

[2] Emole J, Talabi T, Pinilla-Ibarz J (2016). Update on the management of Philadelphia chromosome positive chronic myelogenous leukemia: role of nilotinib. Biologics.

[3] Chakraborty C, Sharma AR, Patra BC, Bhattacharya M, Sharma G, Lee SS (2016). MicroRNAs mediated regulation of MAPK signaling pathways in chronic myeloid leukemia. Oncotarget.

[4] Pantel K, Brakenhoff RH (2004). Dissecting the metastatic cascade. Nature reviews cancer.

[5] Deryugina EI, Quigley JP (2006). Matrix metalloproteinases and tumor metastasis. Cancer and metastasis reviews.

[6] Dahlmann M, Kobelt D, Walther W, Mudduluru G, Stein U (2016). S100A4 in cancer metastasis: Wnt signaling-driven interventions for metastasis restriction. Cancers (Basel).

[7] Guan X (2015). Cancer metastases: challenges and opportunities. Acta pharmaceutica sinica B.

[8] Fingleton B (2005). Matrix metalloproteinases: roles in cancer and metastasis. Frontiers in bioscience.

[9] Visse R, Nagase H (2003). Matrix metalloproteinases and tissue inhibitors of metalloproteinases structure, function, and biochemistry. Circulation research.

[10] Bäck M, Ketelhuth DF, Agewall S (2010). Matrix metalloproteinases in atherothrombosis. Progress in cardiovascular diseases.

[11] Chen PS, Shih YW, Huang HC, Cheng HW (2011). Diosgenin, a steroidal saponin, inhibits migration and invasion of human prostate cancer PC-3 cells by reducing matrix metalloproteinases expression. PLoS one.

[12] González-Arriaga P, Pascual T, García-Alvarez A, Fernández-Somoano A, López-Cima MF, Tardón A (2012). Genetic polymorphisms in MMP 2, 9 and 3 genes modify lung cancer risk and survival. BMC cancer.

[13] Ries C, Loher F, Zang C, Ismair MG, Petrides PE (1999). Matrix metalloproteinase production by bone marrow mononuclear cells from normal individuals and patients with acute and chronic myeloid leukemia or myelodysplastic syndromes. Clinical cancer research.

[14] Blázquez C, Salazar M, Carracedo A, Lorente M, Egia A, González-Feria L, Haro A, Velasco G, Guzmán M (2008). Cannabinoids inhibit glioma cell invasion by down-regulating matrix metalloproteinase-2 expression. Cancer research.

[15] Hermanson DJ, Marnett LJ (2011). Cannabinoids, endocannabinoids, and cancer. Cancer and metastasis reviews.

[16] Velasco G, Hernández-Tiedra S, Dávila D, Lorente M (2016). The use of cannabinoids as anticancer agents. Progress in neuro-psychopharmacology and biological psychiatry.

[17] Murnion B (2015). Medicinal cannabis. Australian prescriber.

[18] Alberich Jordà M, Rayman N, Tas M, Verbakel SE, Battista N, van Lom K, Löwenberg B, Maccarrone M, Delwel R (2004). The peripheral cannabinoid receptor Cb2, frequently expressed on AML blasts, either induces a neutrophilic differentiation block or confers abnormal migration properties in a ligand-dependent manner. Blood.

[19] Ramer R, Bublitz K, Freimuth N, Merkord J, Rohde H, Haustein M, Borchert P, Schmuhl E, Linnebacher M, Hinz B (2012). Cannabidiol inhibits lung cancer cell invasion and metastasis via intercellular adhesion molecule-1. FASEB journal.

[20] Ramer R, Merkord J, Rohde H, Hinz B (2010). Cannabidiol inhibits cancer cell invasion via upregulation of tissue inhibitor of matrix metalloproteinases-1. Biochemical pharmacology.

[21] Hermanson DJ, Gamble-George JC, Marnett LJ, Patel S (2014). Substrate-selective COX-2 inhibition as a novel strategy for therapeutic endocannabinoid augmentation. Trends in pharmacological sciences.

[22] Klein TW, Newton C, Larsen K, Lu L, Perkins I, Nong L, Friedman H (2003). The cannabinoid system and immune modulation. Journal of leukocyte biology.

[23] Shrivastava A, Kuzontkoski PM, Groopman JE, Prasad A (2011). Cannabidiol induces programmed cell death in breast cancer cells by coordinating the cross-talk between apoptosis and autophagy. Molecular cancer therapeutics.

[24] Sarfaraz S, Afaq F, Adhami VM, Mukhtar H (2005). Cannabinoid receptor as a novel target for the treatment of prostate cancer. Cancer research.

[25] Fogli S, Nieri P, Chicca A, Adinolfi B, Mariotti V, Iacopetti P, Breschi MC, Pellegrini S (2006). Cannabinoid derivatives induce cell death in pancreatic MIA PaCa-2 cells via a receptor-independent mechanism. FEBS letters.

[26] Carpi S, Fogli S, Romanini A, Pellegrino M, Adinolfi B, Podestà A, Costa B, Da Pozzo E, Martini C, Breschi MC, Nieri P (2015). AM251 induces apoptosis and G2/M cell cycle arrest in A375 human melanoma cells. Anti-cancer drugs.

[27] Freimuth N, Ramer R, Hinz B (2010). Antitumorigenic effects of cannabinoids beyond apoptosis. The journal of pharmacology and experimental therapeutics.

[28] Endsley MP, Aggarwal N, Isbell MA, Wheelock CE, Hammock BD, Falck JR, Campbell WB, Nithipatikom K (2007). Diverse roles of 2-arachidonoylglycerol in invasion of prostate carcinoma cells: Location, hydrolysis and 12-lipoxygenase metabolism. International journal of cancer.

[29] Xian XS, Park H, Cho YK, Lee IS, Kim SW, Choi MG, Chung IS, Han KH, Park JM (2010). Effect of a synthetic cannabinoid agonist on the proliferation and invasion of gastric cancer cells. Journal of cellular biochemistry.

[30] Farsandaj N, Ghahremani MH, Ostad SN (2012). Role of cannabinoid and vanilloid receptors in invasion of human breast carcinoma cells. Journal of environmental pathology, toxicology and oncology.

[31] Nithipatikom K, Endsley MP, Isbell MA, Falck JR, Iwamoto Y, Hillard CJ, Campbell WB (2004). 2-Arachidonoylglycerol a novel inhibitor of androgen-independent prostate cancer cell invasion. Cancer research.

[32] Preet A, Qamri Z, Nasser MW, Prasad A, Shilo K, Zou X, Groopman JE, Ganju RK (2011). Cannabinoid receptors, CB1 and CB2, as novel targets for inhibition of non–small cell lung cancer growth and metastasis. Cancer prevention research (Phila).

[33] Xu D, Wang J, Zhou Z, He Z, Zhao Q (2015). Cannabinoid WIN55, 212-2 induces cell cycle arrest and inhibits the proliferation and migration of human BEL7402 hepatocellular carcinoma cells. Molecular medicine reports.

[34] Ramer R, Hinz B (2008). Inhibition of cancer cell invasion by cannabinoids via increased expression of tissue inhibitor of matrix metalloproteinases-1. Journal of the national cancer institute.

[35] Malfitano AM, Ciaglia E, Gangemi G, Gazzerro P, Laezza C, Bifulco M (2011). Update on the endocannabinoid system as an anticancer target. Expert opinion on therapeutic targets.

[36] Bifulco M, Laezza C, Portella G, Vitale M, Orlando P, De Petrocellis L, Di Marzo V (2001). Control by the endogenous cannabinoid system of ras oncogene-dependent tumor growth. FASEB journal.

[37] Ryberg E, Vu HK, Larsson N, Groblewski T, Hjorth S, Elebring T, Sjögren S, Greasley PJ (2005). Identification and characterisation of a novel splice variant of the human CB1 receptor. FEBS letters.

[38] Michalski CW, Oti FE, Erkan M, Sauliunaite D, Bergmann F, Pacher P, Batkai S, Müller MW, Giese NA, Friess H, Kleeff J (2008). Cannabinoids in pancreatic cancer: correlation with survival and pain. International journal of cancer.

